# Neurofilaments as Biomarkers for Amyotrophic Lateral Sclerosis: A Systematic Review and Meta-Analysis

**DOI:** 10.1371/journal.pone.0164625

**Published:** 2016-10-12

**Authors:** Zhouwei Xu, Robert David Henderson, Michael David, Pamela Ann McCombe

**Affiliations:** 1 Centre for Clinical Research, the University of Queensland, Brisbane, Queensland, Australia; 2 Department of Neurology, Royal Brisbane & Women’s Hospital, Brisbane, Queensland, Australia; 3 School of Public Health, the University of Queensland, Brisbane, Queensland, Australia; Institute of Health Science, CHINA

## Abstract

**Background:**

To allow early diagnosis and monitoring of disease progression, there is a need for biomarkers in amyotrophic lateral sclerosis (ALS). Neurofilaments (NF) are emerging protein biomarkers in other neurological diseases, and are of possible use in ALS.

**Objective:**

The aim of this study is to evaluate the utility of NF levels as blood or cerebrospinal fluid (CSF) biomarker in patients with ALS.

**Methods:**

A systematic search of Pubmed, Embase and Scopus was performed. Methodological quality assessment was applied to refine the final search results. Meta-analysis of the data was performed.

**Results:**

Level of NF heavy chain and light chains were significantly elevated in the CSF of ALS patients compared to healthy controls/controls without parenchymal central nervous system (CNS) involvement and ALS mimic disease patients. NF light chain level in CSF was higher in ALS patients than in neurological patients with CNS involvement (SMD = 1.352, P = 0.01). NF light chain concentration in blood was higher in ALS patients than healthy controls/controls without CNS involvement (SMD = 1.448, P<0.0001). NF heavy chain levels in CSF were negatively correlated disease duration and ALSFRS-R ((r = -0.447, P<0.0001; r = -0.486, P<0.0001). NF light chain levels in CSF were negatively correlated with disease duration (r = -0.273, P = 0.011).

**Conclusion:**

NF heavy and light chain levels have potential use as a marker of neural degeneration in ALS, but are not specific for the disease, and are more likely to be used as measures of disease progression.

## Introduction

Neurofilaments (NF) are intermediate filaments that are major components of neuronal cytoskeleton. NF can be divided according to the observed molecular weight into NF light chain (68 kda), NF intermediate chain (160 kda) and NF heavy chain (205kda) [1]. The three different NFs share a conserved alpha-helical rod domain, but differ in the head and tail domains [2]. NFM and NFH are always phosphorylated [3]. NFL is susceptible to protease degradation, while NFH, which is phosphorylated, can resist protein degradation [4]. Damage to axons of the central nervous system (CNS) or peripheral nervous system (PNS)could release the NF, which would then appear in the cerebrospinal fluid (CSF) and the blood stream, where NF can be detected with techniques such as enzyme linked immunosorbent assay (Elisa), western blot and mass spectrometry[5, 6].

Amyotrophic lateral sclerosis (ALS) is a fatal neurodegenerative disease, which involves progressive loss of both upper and lower motor neurons. The incidence of ALS is about 1–2 per 100,000 [7]. Almost 90% of the cases of ALS are sporadic and 5–10% of the cases are familial [8]. Generally, the median survival of patients from symptom onset is about 2–3 years [9] and the cause of the death is respiratory failure. However, there is heterogeneity of disease survival, and approximately 10% of patients survive for more than 10 years [10]. Analysis of spinal cord tissue has suggested abnormal NFH subunit accumulation in neuronal perikarya and spheroids in ALS patients compared with control [11].

There is a need for a biomarker in ALS, for use in diagnosis, prognosis and in clinical trials. Katz et al. defined biomarkers as a "objectively measured and evaluated parameters for indication of pathological processes, disease progression or response to pharmacological interventions"[12]. Various bodily fluids have been used for biomarkers, including blood, CSF, urine and saliva. Among these biofluids, blood and CSF have differing advantages. CSF is expected to contain proteins from degenerating neurons because of its direct contact with the CNS, so is suitable for the study of CNS disease. Blood samples have a less invasive process of collection, which is much more acceptable for patients. Biomarkers that enter the CSF will eventually drain into the veins, so blood samples will include protein released from degenerating neurons. The disadvantage of studying blood is its complexity, as blood contains many different proteins.

The aim of this systematic review and meta-analysis is to investigate whether NF levels in blood or CSF could be a reliable biomarker for amyotrophic lateral sclerosis, either in distinguishing patients from controls or as markers monitoring disease progression or predicting prognosis. The preferred reporting items for systematic review and meta-analysis (PRISMA) checklist was shown in the S1 File[13]

## Methods

### 1. Search strategy and key words

A systematic search was conducted in Pubmed, Embase, Scopus and Medline, with the latest date of search being 20^th^ May 2016.

The search strategy in Pubmed is as follows:

((((((((((("Blood"[Mesh]) OR (((("Cerebrospinal Fluid"[Mesh]) OR "Cerebrospinal Fluid") OR "Cerebrospinal Fluids")))) OR blood)) AND "Amyotrophic Lateral Sclerosis"[Mesh]) AND neurofilament*) AND "Humans"[Mesh]) AND English[lang])) AND (((((("Biomarkers"[Mesh]) OR biomarker*))))))

The search strategy in Embase is as follows:

'amyotrophic lateral sclerosis'/exp AND ('biological marker'/exp OR biomarker OR biomarkers) AND ('neurofilament'/exp OR neurofilament OR neurofilaments) AND ('blood'/exp OR ‘cerebrospinal fluid'/exp OR ‘cerebrospinal fluid’ OR 'cerebrospinal fluids') AND 'human'/de

The search strategy in Scopus is as follows:

(TITLE-ABS-KEY(“Amyotrophic Lateral Sclerosis”))and ((TITLE-ABS-KEY(blood) OR TITLE-ABS-KEY(“Cerebrospinal Fluid”)OR TITLE-ABS-KEY(“Cerebrospinal Fluids”))) and ((TITLE-ABS-KEY(biomarker) OR TITLE-ABS-KEY(biomarkers))) and ((TITLE-ABS-KEY(neurofilament) OR TITLE-ABS-KEY(neurofilaments)))AND(LIMIT-TO(EXACTKEYWORD,"Human")) AND (LIMIT-TO(LANGUAGE,"English"))

### 2. Selection criteria

The inclusion criteria are as follows:

1. The study must be carried out in humans more than 18 years of age.
2. All the subjects with ALS must be diagnosed with the EL Escorial criteria or revised El Escorial [14] or Awaji [15] criteria.
3. A demographic description of the patients must be provided.
4. The study must use NF as a biomarker in the blood or CSF to differentiate ALS patients from a control group or to distinguish subgroups of ALS patients.
5. The study must provide clear description of the time point and method for collection of blood and CSF.
6. The study must provide a full description of the method used to measure NF levels.
7. The study must be the original paper rather than abstracts, posters or reviews.

The exclusion criteria are as follows:

1. The subjects were younger than 18 years.
2. The ALS subjects had additional neurological disorders or pathologic changes such as brain tumour, epilepsy or brain injury.
3. The number of subjects was less than 5.
4. Studies without healthy controls or without disease control.
5. The study was not published in English.
6. Studies with missing data such as details about the demographic information and details of method used for NF detection.
7. The papers are abstracts or posters or reviews

### 3. Assessment of methodological quality

A total of 20 papers were selected for the final systematic review. The original extracted data on NF levels is summarized in S1, S2, S3 and S4 Datasets. We used the QUADAS-2 (Quality Assessment of Diagnostic Accuracy Studies) criteria to assess the 20 papers selected for systematic review[16]. This tool is made up of 4 key domains which assessed the risk of bias covering patient selection, index test, reference standard and flow and timing respectively. The first three domains were also assessed the concerns in terms of applicability. The assessment was done independently by two authors (ZX and PM). If there was disagreement, the third author RH was consulted to resolve the disagreement. The final results of quality assessment of the 20 papers and the proportion of high quality and low quality papers were summarized in S1 Table.

### 4. Data synthesis and statistics

We attempted to obtain the original data by contacting the authors of the selected papers, but this was not available. Comprehensive meta-analysis software V 2.0 (Biostat, USA) was used to perform the final data combination and meta-analysis. When the mean value, standard deviation (SD), correlation coefficient and size of cohort were not available, a series of formulas, as described by Hozo et al. and Wan et al. [17, 18] was utilized to estimate the sample mean and standard deviation from the published sample size, median, range or inter-quartile range. If the median was the only value provided, the study could not be included into the meta-analysis.

The studies used a variety of disease controls. To deal with this heterogeneity, we subdivided the disease controls into three groups; 1) healthy control/controls without parenchymal involvement of the CNS, 2) ALS mimics and 3) controls with neurological disease with parenchymal CNS involvement. The controls without parenchymal CNS involvement had a range of conditions including Guillain-Barré syndrome, tension headache, back pain, normal pressure hydrocephalus, facial palsy and polyneuropathy.

When doing the meta-analysis, as required by the Cochrane handbook for systematic reviews of Interventions[19], we used standardized mean difference to combine the overall effect on the basis of the mean± SD value from each study. We used Fisher’s Z test to combine the overall effect on the basis of the correlation coefficient and sample size. P value less than 0.05 was considered as significant. If the I^2^ statistics of the heterogeneity of the studies was less than 40%, the fixed effect meta-analysis model was chosen. If the I^2^ statistics was more than 50%, the random effect model was applied [19].

The results of the meta-analysis of NF concentration difference between the ALS group and the control groups are illustrated as forest plots that show the standardized mean difference (SMD) between the two groups. The results of the meta-analysis of correlations between NF and disease duration or progression are also illustrated as forest plots that show the correlation coefficient between the two variables.

## Results

### 1. Study characteristics

**Fig 1:**
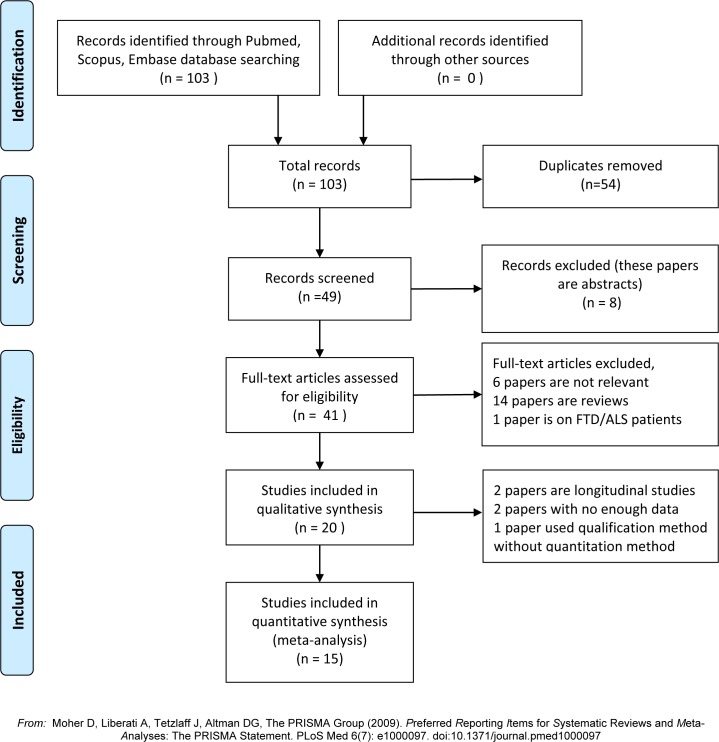
PRISMA Flow diagram. Flow diagram of systematic search in the 3 databases. After removal of duplicates, reviews and quality control, 20 papers were suitable for analysis.

The total number of papers obtained from the search was 49. The flow diagram based on PRISMA is shown in Fig 1[13]. S2 Table shows the details of 20 papers assessed as being suitable for the systematic review. The details of the controls in every paper are also summarized in the S2 Table. The 20 papers are categorized according to the biofluids that were sampled. The summarized results are shown in the S3 Table. There were more studies of NFH than NFL. Because the NF heavy chain is phosphorylated, all the detection methods were targeted to phosphorylated NFH (pNFH). The terms pNFH and NFH are used interchangeably in the literature. The summary of the results of the meta-analysis are shown in Table 1.

**Table 1:** Summary of the meta-analyses.

	ALS v HC/non CNS disease			ALS v mimic disease			ALS v CNS disease		
	No of studies	Total no of subjects (patients/ controls)	P value of meta-analysis	No of studies	Total no of subjects (patients/ control)	P value of meta-analysis	No of studies	Total no of subjects (patients/ controls)	P value of meta-analysis
NFH in CSF	4 studies (5 cohorts)	443/267	P<0.0001	2 studies	251/100	P = 0.013	6 studies(11 cohorts)	468/329	P = 0.075
NFH in blood	2 studies	117/78	P = 0.057						
NFL in CSF	6 studies(7 cohorts)	463/214	P<0.0001	2 studies	250/99	P<0.0001	5 studies	398/405	P = 0.001
NFL in blood	3 studies(5 cohorts)	202/277	P<0.0001						

### 2. NFH levels in CSF

#### ALS versus healthy controls/controls without parenchymal CNS involvement

**Fig 2:**
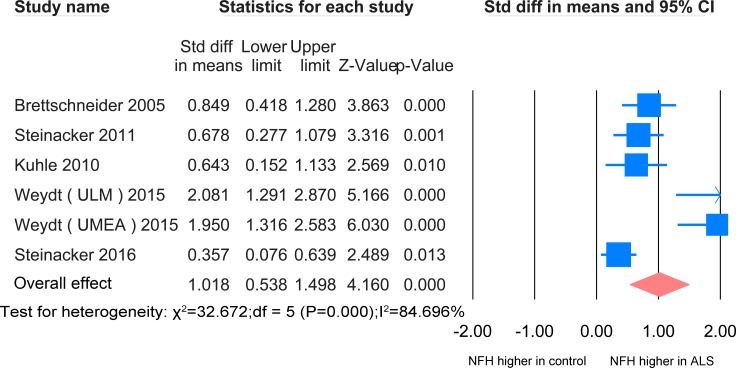
NFH CSF ALS-healthy. Meta-analysis of NFH levels in CSF between ALS patients and healthy controls/controls without parenchymal CNS disease. The random effect model was used. There was a highly significant difference between the two groups (P <0.0001).

In CSF, the NFH meta-analysis between ALS patients and healthy controls/controls without parenchymal CNS involvement was based on 5 studies [20–24], all using ELISA, including 443 ALS patients and 267 healthy controls (Fig 2). The level of NFH in ALS patients is significantly higher than that of the controls (SMD = 1.018, P <0.0001). Another paper used Western blot analysis and identified one unique pNFH in the CSF of ALS patients, which is absent in the CSF of healthy controls [25].

#### ALS versus ALS mimics disease

**Fig 3:**
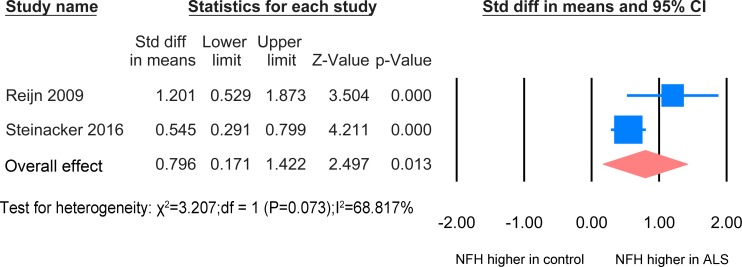
NFH CSF ALS-mimics. Meta-analysis of NFH levels in CSF between ALS patients and ALS mimics. The random effect was applied. There was a significant difference between the two groups (P = 0.013).

In CSF, the NFH meta-analysis between ALS patients and ALS mimic disease controls was based on 2 studies [24, 26] including 251 patients and 100 ALS mimic disease controls on basis of ELISA technology (Fig 3). The standardized mean difference is significant (SMD = 0.796, P = 0.013).

#### ALS versus other neurological diseases with CNS involvement

**Fig 4:**
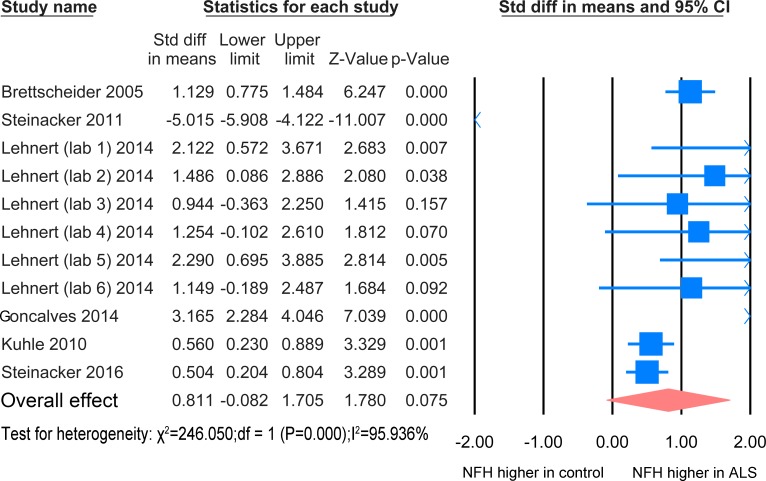
NFH CSF ALS-CNS disease. Meta-analysis of NFH levels in CSF between ALS patients and other neurological disease with CNS involvement. The random effect model was used. There is no significant difference between the two groups (P = 0.075).

The NFH meta-analysis between ALS patients and patients with other neurological disease with CNS involvement was based on 6 studies [20–22, 24, 27, 28] including 329 disease controls and 468 ALS patients (Fig 4). There was no significant difference between the two groups (P = 0.075).

Higher levels of pNFH^SMI35^ were reported in patients with upper motor neuron dominant disease than in those with typical ALS [20]. Higher levels were also reported in symptomatic carriers of causative mutations than asymptomatic carriers [23]. Another paper suggested that the ratio of CSF pNFH/complement C3 could be used to discriminate the ALS patients and disease control [29].

### 3. NFH levels in Blood

#### ALS versus healthy controls/controls without parenchymal CNS involvement

**Fig 5:**
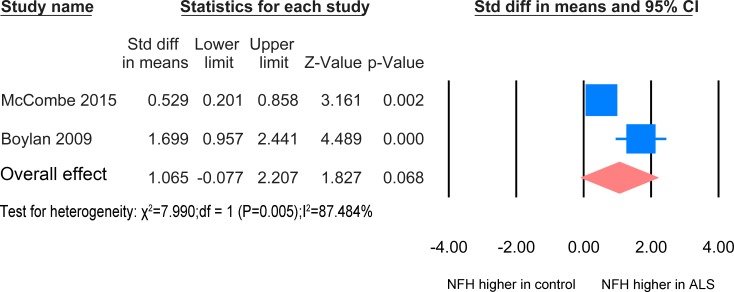
NFH Blood ALS-healthy. Meta-analysis of NFH levels in blood between ALS patients and healthy controls /controls without parenchymal CNS disease. The random effect model was applied. There is no significant difference between the two groups (P = 0.068).

The result for the NFH meta-analysis between healthy controls/controls without parenchymal CNS involvement and ALS patients in blood was based on 2 studies [30, 31] including 78 healthy controls and 117 patients (Fig 5). The concentration of NFH in the ALS patients group was not significantly increased relative to the healthy controls/ without CNS involvement (P = 0.057). Another paper [29] also found an increased level of NFH in ALS compared to healthy controls and other neurological disease, but there was insufficient data for inclusion in the meta-analysis.

There was no significant difference in levels of NFH between patients treated with Riluzole and those without [30]. Bulbar onset patients were reported to have elevated concentration of NFH in plasma compared with spinal onset patients [32]. There is correlation of pNFH between CSF and blood levels of NFH [29]. Two papers reported that the concentration of NFH in the blood showed an initial rise but later the levels fell [30, 32].

### 4. NFL levels in CSF

#### ALS versus healthy controls/controls without parenchymal CNS involvement

**Fig 6:**
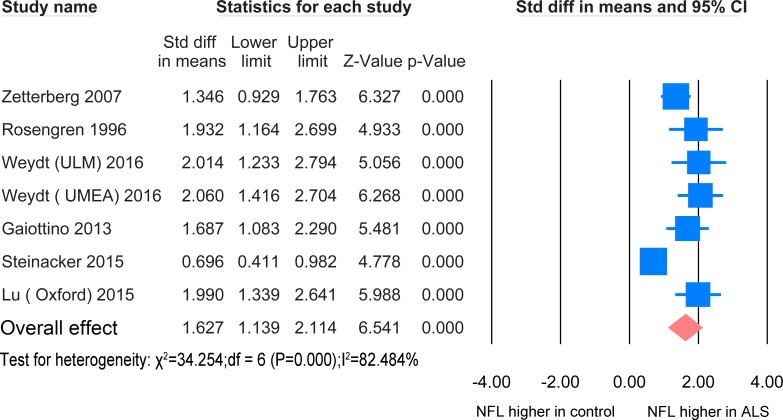
NFL CSF ALS-healthy. Meta-analysis of NFL levels in CSF between ALS patients and healthy controls/controls without parenchymal CNS disease. The random effect model was applied. There is significant difference between the two groups (P <0.0001).

The NFL meta-analysis between ALS patients and healthy controls/controls without parenchymal CNS involvement in CSF was based on 6 studies [23, 24, 33–36] including 463 ALS patients and 214 healthy controls (Fig 6). The standardized mean difference was significantly increased in comparison with the controls (SMD = 1.627, P <0.0001).

#### ALS versus ALS mimics disease

**Fig 7:**
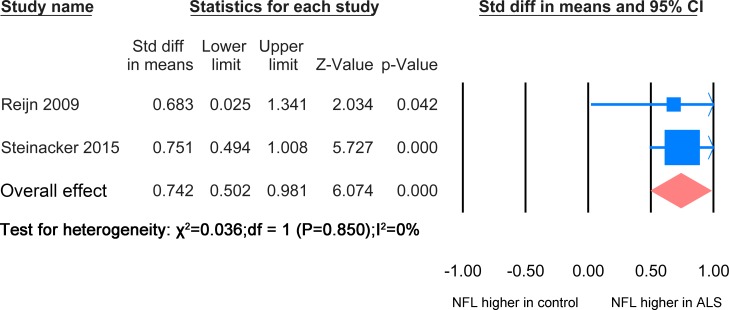
NFL ALS-mimic. Meta-analysis of NFL levels in CSF between ALS patients and ALS mimics. Because the I^2^ = 0, the fixed effect model was applied. There is significant difference between the two groups (P<0.0001).

The NFL meta-analysis between ALS patients and mimic disease control was based on 2 studies [24, 26] including 250 ALS patients and 99 ALS mimic disease patients (Fig 7). The level of NFL in ALS mimic disease was significantly lower than the ALS patients group (SMD = 0.742, P <0.0001).

#### ALS versus other neurological diseases with CNS involvement

**Fig 8:**
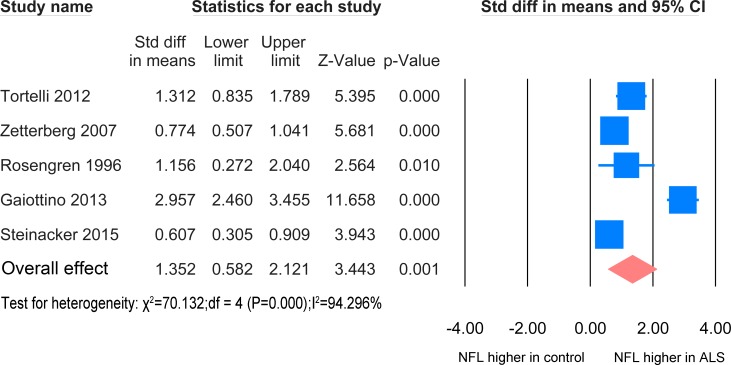
NFL CSF ALS-CNS disease. Meta-analysis of NFL levels in CSF between ALS patients and other neurological disease with CNS involvement. The random effect model was applied. There is significant difference between the two groups (P = 0.001).

The meta-analysis of ALS patients and disease control with CNS involvement in CSF samples was based on 5 studies [24, 33–35, 37] including 398 ALS patients and 405 disease controls. (Fig 8), A significant elevation of NFL was found in the ALS patients (SMD = 1.625, P = 0.001).

No difference of NFL level in the CSF was found between different phenotypes and no correlation was found between NFL level in the CSF and EMG features of lower motor neuron denervation [37]. The level of NFL in the CSF of symptomatic ALS gene mutation carriers was significantly greater than that of asymptomatic ALS mutation carriers [23] and patients carrying a SOD1 mutation have a lower concentration of NFL than those without [34].

### 5. NFL levels in blood

#### ALS versus healthy controls/controls without parenchymal CNS involvement

**Fig 9:**
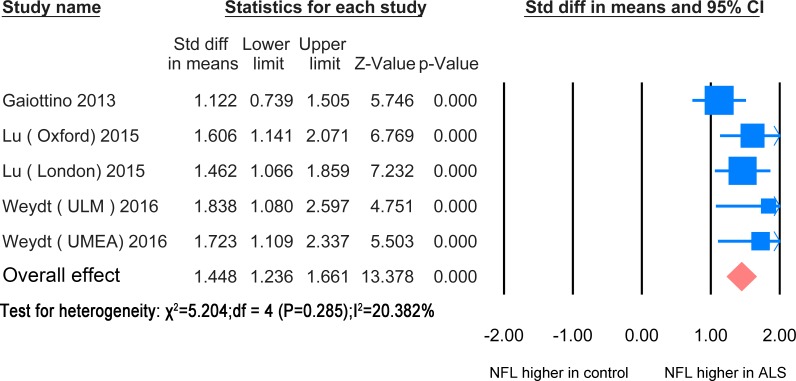
NFL blood ALS-healthy. Meta-analysis of NFL levels in blood between ALS patients and healthy controls/controls without parenchymal CNS disease. The fixed effect model was applied. There is significant difference between the two groups (P< 0.0001).

In blood, the NFL meta-analysis between ALS patients and healthy controls /controls without CNS involvement was based on 3 studies [23, 35, 36] including 277 healthy controls and 202 ALS patients (Fig 9). The standardized mean difference between the two groups was statistically significant (SMD = 1.448, P<0.0001). The papers used ELISA except for one which used elecrochemiluminescence (ECL) based immune assay [35].

Symptomatic ALS gene mutation carriers had significantly increased blood levels of NFL compared asymptomatic carriers of causative mutations.[23]. There were no differences in the blood NFL level between ALS patients treated with Riluzole and those without [30]. Three papers found that there was a high correlation of NFL levels between CSF and serum and/or plasma [23, 35, 36].

### 6. Correlation with the measures of disease

In the papers selected for analysis, there have been attempts to determine whether levels of NF are related to the measures of disease. This has been done by examining the relationship of NF with the disease duration, disease progression measured by revised ALS functional rating scale (ALSFRS-R) [38] and survival time. There was considerable heterogeneity among the statistic approaches used in the different papers. So only limited meta-analysis was possible.

#### Correlation with disease duration

**Fig 10:**
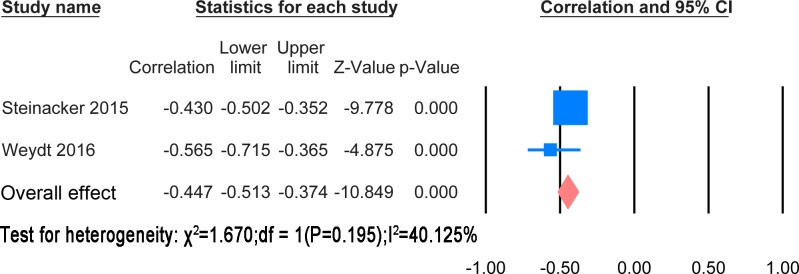
CSF NFH and disease duration. Meta-analysis of correlation between CSF NFH and disease duration. The fixed effect model was applied. There is significant negative correlation between two variables (P< 0.0001).

**Fig 11:**
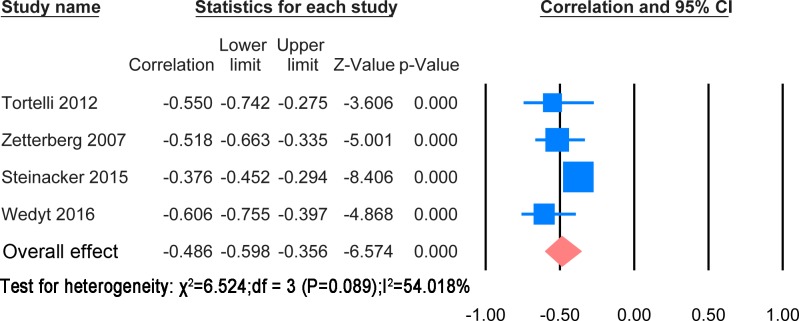
CSF NFL and disease duration. Meta-analysis of correlation between CSF NFL and disease duration. The random effect model was applied. There is significant negative correlation between two variables (P< 0.0001).

The extracted results and statistic methods of the correlation with disease duration were summarised in S4 Table. The majority of papers showed a negative correlation of disease duration with NF levels. There was heterogeneity in the statistical methods. However, a meta-analysis was possible for correlation of CSF NFH and NFL levels with disease duration. These are shown in Figs 10 and 11 respectively.

The correlation meta-analysis between CSF NFH and disease duration was based on 2 studies [23, 24] including 516 patients. The overall correlation coefficient was significant (r = -0.447, P<0.0001). The correlation meta-analysis between CSF NFL and disease during was based on 4 studies [23, 24, 34, 37] including 622 patients. The overall correlation coefficient was significant (r = -0.486, P<0.0001).

#### Correlation with disease duration

**Fig 12:**
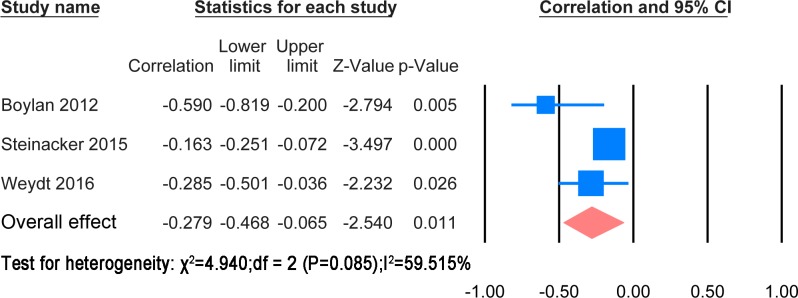
CSF NFH and ALSFRS-R. Meta-analysis of correlation between CSF NFH and ALSFRS-R. The random effect model was applied. There is significant negative correlation between two variables (P = 0.011).

**Fig 13:**
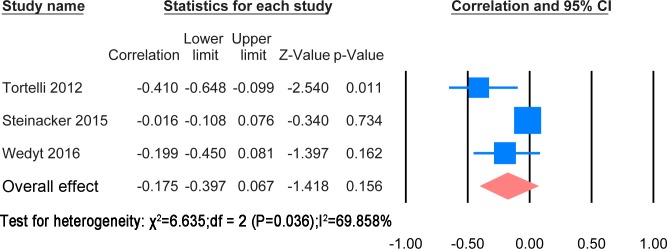
CSF NFL and ALSFRS-R. Meta-analysis of correlation between CSF NFL and ALSFRS-R. The random effect model was applied. There is no significant negative correlation between two variables (P = 0.156).

The extracted results and statistic methods of the correlation with disease progression were summarised in S4 Table. The papers used different statistic method to find the correlation with ALSFRS-R or the rate of change of ALSFRS-R. Cross sectional correlation of NF levels with ALSFRS-R scores and comparison between patients with rapid progression and ones with slow progression were also mentioned in the papers. A meta-analysis was possible for correlation of CSF NFH and NFL levels with ALSFRS-R score. These are shown in Figs 12 and 13 respectively.

The correlation meta-analysis between CSF NFH and ALSFRS-R score was based on 3 studies [23, 24, 32] including 536 patients. The overall correlation coefficient was significant (r = -0.273, P = 0.011). The correlation meta-analysis between CSF NFL and ALSFRS-R was based on 3 studies [23, 24, 37] including 543 patients. The overall correlation coefficient was not significant (r = -0.447, P = 0.156). One study suggested using the ratio CSF pNFHSMI35/CSF sAPP α—β to monitor disease progression [21]. Another paper discovered that the CSF NFL was correlated with spreading from localized to generalized weakness [39].

#### Correlation with survival time or prognosis

The extracted results and statistic methods of the correlation with disease progression were summarised in S4 Table. Due to the considerable heterogeneity of statistic method. The meta-analysis of correlation between NF and survival time was impossible. Several papers found that the level of NF or the rate of rise of NF was negatively correlated with survival time or increasing odds ratio of death in both CSF and blood[24, 30, 32, 34, 36, 37]. Only one paper pointed out that no correlation was found between cumulative mortality and base line pNFH[31].

### 7. Sensitivity and Specificity of NF

Some of the papers provided and used receiver operating characteristic (ROC) to analyse the sensitivity and sensitivity of the NF levels in distinguishing ALS from the various control groups. The extracted results of ROC were shown in the S5 Table. Different optimal cut-off values were used among the papers, the sensitivity of ROC analysis ranges from 0.71 to 0.91. The specificity of ROC analysis ranges from 0.64 to 0.95. Area under curve (AUC) ranges from 0.77 to 0.9987. The ROC analysis on blood NF was very few.

## Discussion

Here we have analysed the studies of NFH and NFL as biomarkers of ALS. The studies used different controls, so we grouped the controls into healthy controls/ without parenchymal CNS disease, ALS mimic diseases and CNS parenchymal diseases. In CSF, both NFL and NFH levels were significantly greater in ALS than in healthy controls/patients without parenchymal CNS disease, while for blood, NFL levels showed a highly significant difference. For NFH, the number of subjects was lower than for CSF, and the difference was not statistically significant. Comparing ALS with other CNS disease, and comparing ALS with ALS mimic disease, only CSF was studied. CSF NFL and NFH levels were significantly greater in ALS than ALS mimic disease. CSF NFL levels, but not CSF NFH levels, were significantly greater in ALS than the other CNS diseases that were tested.

Before doing the meta-analysis, we attempted to get all the original data from the author of every paper. However it was not possible due to the confidentiality or other various reasons from the authors. So we used a series of formulas to infer and calculate the mean and SD from the published sample size, median, range or inter-quartile range. These formulas were widely acknowledged and used in other meta-analysis [40, 41].

The finding that NFH levels are higher in ALS than in healthy controls/non CNS parenchymal disease is consistent with the view that damage to axons releases NF. There was a significant difference between CSF NFL and the CNS diseases used as controls, but it must be noted that elevation of NF levels is not specific for ALS and has been reported in a number of other CNS diseases [42, 43] [2, 44–47]. For example, levels of NF in subarachnoid haemorrhage were much higher than in ALS patients [22] and similar results were also observed in the other studies [48, 49].

In the meta-analysis, we included GBS data in the control group without parenchymal CNS involvement. However, two researchers reported that the NFH or NFL in the CSF of GBS patients was higher than in the other neurological disease such as AD and PD with CNS involvement but less than ALS patients [22, 35]. NF are found in peripheral as well as central nervous system axons, and can also be released by peripheral axonal degeneration.

Given that raised NF levels are not specific for ALS, the most useful role of NF levels is likely to be in monitoring duration, progression or prognosis of disease. So we did meta-analyses about the correlation between CSF NFH or CSF NFL with ALSFRS-R score and disease duration. The final results showed that CSF NFH had a significant negative association with both disease duration and progression. Significant correlation was also found between CSF NFL and disease duration. Conversely no significant relationship was found between CSF NFL and disease progression. Worse prognosis or high odds ratio of death was also closely related with high baseline NF in most of the papers. However the NFL role in these fields still need to be verified and replicated with larger cohort by using same or standardized measurement scale in these three fields. There were some attempts to correlate NF levels with disease phenotype, but these were few.

To advance the field of NF as a biomarker, there needs to be standardization of the techniques that are used. In the papers reviewed here, ELISA, electrochemiluminescence (ECL) based immunoassay and western blot were used, with ELISA being used most commonly. One paper pointed out the traditional ELISA kit method was more sensitive than the ECL based immunoassay [50]. The versions of ELISA used in these studies were more sensitive than earlier methods [6, 51, 52]. The minimum limit of NFL ELISA kit used by the relevant papers ranges from 30 ng/ml to 125ng/ml. The phenomenon of the "hook effect" is an issue with measurements of NFH. This effect occurs with neurofilaments aggregation when blood samples are not diluted. [53], preventing the accurate quantification by immunoassay. This phenomenon was not observed in NFL in blood or with either NFL or NFH in CSF. As to the specificity and sensitivity of NF in the ALS patients, they not only depended on the detection methods as mentioned above, but also depends on the optimal cut-off and what other diseases to be differentiated from ALS. Neurological disease with more axon injury involvement was less easy to differentiate in comparison with healthy people.

In summary, a series of meta-analyses have shown that the NFH and NFL in CSF sample can be used to discriminate the ALS patients from the healthy people, and ALS mimics disease. NFL chain in CSF and blood sample can also be used to differentiate the ALS patients from the other neurological disease and healthy people respectively. There is promising evidence that NF levels could be used to monitor disease progression but this needs further work.

## Supporting Information

S1 Dataset CSF NFH original extracted data. (XLSX)

S2 Dataset CSF NFL original extracted data. (XLSX)

S3 Dataset Blood NFH original extracted data. (XLSX)

S4 Dataset Blood NFL original extracted data. (XLSX)

S1 File PRISMA checklist. (DOC)

S1 Table Results of Quadas-2 quality assessment. (DOCX)

S2 Table Summarized details of the papers. (DOCX)

S3 Table Summary of the papers according to the biofluid. (DOCX)

S4 Table Summary of studies correlated with disease duration, progression and survival time. (DOCX)

S5 Table Summary of specificity and sensitivity of the papers. (DOCX)
