# Supplementary material for: Neurofilaments as Biomarkers for Amyotrophic Lateral Sclerosis: A Systematic Review and Meta-Analysis
Source: PLoS One. 2016 Oct 12;11(10):e0164625. doi: 10.1371/journal.pone.0164625 (PMC5061412; doi:10.1371/journal.pone.0164625)

The results of Quadas-2

| **Study** | **RISK OF BIAS** | | | | **APPLICABILITY CONCERNS** | | |
| --- | --- | --- | --- | --- | --- | --- | --- |
|  | **PATIENT SELECTION** | **INDEX TEST** | **REFERENCE STANDARD** | **FLOW AND TIMING** | **PATIENT SELECTION** | **INDEX TEST** | **REFERENCE STANDARD** |
| **Boylan 2009** | ☺ | ☺ | ☺ | ☺ | ☺ | ☺ | ☺ |
| **Boylan 2013** | ☺ | ☺ | ☺ | ☺ | ☺ | ☺ | ☺ |
| **Brettschneider 2005** | ☺ | ☺ | ☺ | ☺ | ☺ | ☺ | ☺ |
| **Gaiottino 2013** | ☺ | ☺ | ☺ | ☺ | ☺ | ☺ | ☺ |
| **Ganesalingam 2011** | ☺ | ☺ | ☺ | ☺ | ☺ | ☺ | ☺ |
| **Ganesalingam 2013** | ☺ | ☺ | ☺ | ☺ | ☺ | ☺ | ☺ |
| **Goncalves 2014** | ☺ | ☺ | ☺ | ☺ | ☺ | ☺ | ☺ |
| **Kuhle 2010** | ☺ | ☺ | ? | ☺ | ☺ | ? | ☺ |
| **Lehnert 2016** | ☺ | ☺ | ☺ | ☺ | ☺ | ☺ | ☺ |
| **Lu 2015** | ☺ | ☺ | ☺ | ☺ | ☺ | ☺ | ☺ |
| **McCombe 2015** | ☺ | ☺ | ☺ | ☺ | ☺ | ☺ | ☺ |
| **Mendonca 2011** | ☺ | ☹ | ☺ | ☺ | ☺ | ☹ | ☺ |
| **Reijn 2009** | ☺ | ☺ | ☺ | ☺ | ☺ | ☺ | ☺ |
| **Rosengren 1996** | ☺ | ☺ | ☺ | ☺ | ☺ | ☺ | ☺ |
| **Steinacker 2011** | ☺ | ☺ | ☺ | ☺ | ☺ | ☺ | ☺ |
| **Steinacker 2015** | ☺ | ☺ | ☺ | ☺ | ☺ | ☺ | ☺ |
| **Tortelli 2012** | ☺ | ☺ | ☺ | ☺ | ☺ | ☺ | ☺ |
| **Tortelli 2014** | ☺ | ☺ | ☺ | ? | ☺ | ☺ | ☺ |
| **Weydt 2016** | ☺ | ☺ | ☺ | ? | ☺ | ☺ | ☺ |
| **Zettergerg 2007** | ☺ | ☺ | ☺ | ☺ | ☺ | ☺ | ☺ |

☺Low Risk ☹High Risk ? Unclear Risk

The proportion of the quality assessment


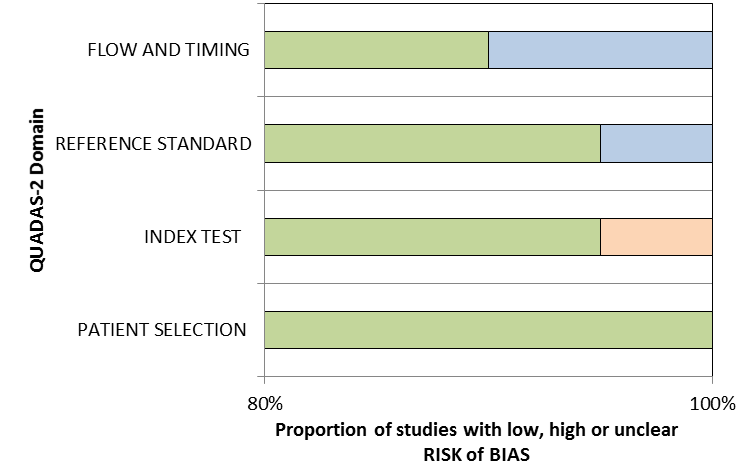

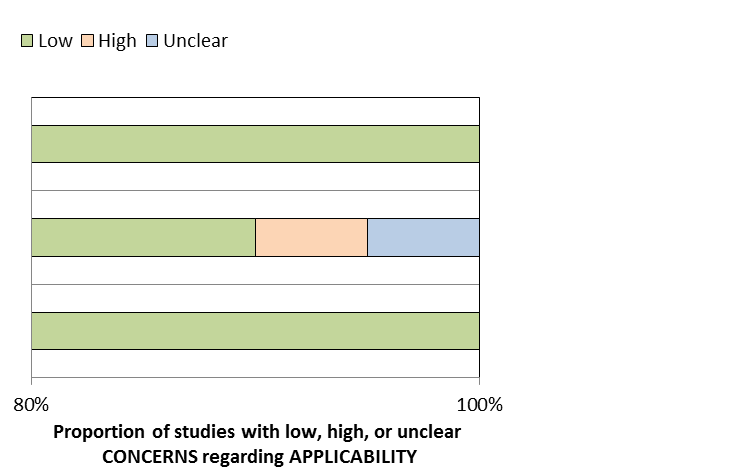

Supplement: S1 Table — (DOCX) [file pone.0164625.s006.docx]
