# Supplementary material for: Neurofilaments as Biomarkers for Amyotrophic Lateral Sclerosis: A Systematic Review and Meta-Analysis
Source: PLoS One. 2016 Oct 12;11(10):e0164625. doi: 10.1371/journal.pone.0164625 (PMC5061412; doi:10.1371/journal.pone.0164625)
Supplement: S2 Table — (DOCX) [file pone.0164625.s007.docx]

Summary of 20 papers selected for meta-analysis

| **Paper** | **NF tested** | **CSF** | **Blood** | **Excluded from meta-analysis, included in systematic review** | **Used for meta-analysis** | | | |
| --- | --- | --- | --- | --- | --- | --- | --- | --- |
|  |  |  |  |  | **Controls used** | | | |
|  |  |  |  |  | **Healthy control** | **Non CNS parenchymal disease** | **ALS mimic disease** | **CNS parenchymal disease** |
| Boylan 2009[[19](#_ENREF_19)] | NFH | No | Yes |  | Yes | No | No | No |
| Boylan 2013[[20](#_ENREF_20)] | NFH | Yes | No | longitudinal study, no controls | N/A | N/A | N/A | N/A |
| Brettschneider 2005[[21](#_ENREF_21)] | NFH | Yes | No |  | No | Tension headache | No | Alzheimer’s disease |
| Gaiottino 2013[[22](#_ENREF_22)] | NFL | Yes | Yes |  | Yes | Tension headache, lower back pain, psychiatric disorders, nonspecific symptoms without neurological explanation, Guillain-Barré syndrome | No | Alzheimer’s disease |
| Ganesalingam 2011[[23](#_ENREF_23)] | NFH | Yes | Yes | insufficient data | Yes | Migraine, Conversion disorder, Normal pressure  hydrocephalus | Yes | Multiple Sclerosis, Frontotemporal  lobar degeneration without motor involvement, Alzheimer’s  Disease, Parkinson’s Disease, Spinocerebellar Ataxia, Neoplasia, Inflammatory  Conditions, infectious  diseases |
| Ganesalingam 2013[[24](#_ENREF_24)] | NFH | Yes | No | insufficient data | No | Benign headache, normal pressure hydrocephalus | Yes | Parkinson ’ s and related syndromes, multiple sclerosis, other neuroinflammatory disorders |
| Goncalves 2014[[25](#_ENREF_25)] | NFH | Yes | No |  | No | No | No | Chronic inflammatory demyelinating neuropathy, hereditary demyelinating polyneuropathy, diabetic neuropathy, probable brachial plexitis, undetermined neuropathies, myelitis , headache, normal pressure hydrocephalus, undetermined spinal cord lesion |
| Kuhle 2010[[26](#_ENREF_26)] | NFH | Yes | No |  | No | Tension type headache, lower back pain ,psychiatric disorders, miscellaneous diseases without neurological explanation, Guillain-Barré syndrome | No | multiple sclerosis,  Mild cognitive impairment/ Alzheimer’s  Disease, subarachnoid haemorrhage |
| Lehnert 2016[[27](#_ENREF_27)] | NFH | Yes | No |  | No | No | No | demyelinating neuropathy, myelitis, stroke, migraine, other headache, ,aneurism, neuropathy, hepatic encephalopathy, sensory TIA, parkinsonism, mild cognitive impairment ,ischaemic optic neuropathy, cerebrovascular disease, somatisation disorder ,hyperreflexia |
| Lu 2015[[28](#_ENREF_28)] | NFL | Yes | Yes |  | Yes | No | No | No |
| McCombe 2015[[29](#_ENREF_29)] | NFH | No | Yes |  | Yes | No | No | No |
| Mendonca 2011[[30](#_ENREF_30)] | NFH | Yes | No | using western blot to measure NF | No | Headache | No | No |
| Reijn 2009[[31](#_ENREF_31)] | NFH and NFL | Yes | No |  | No | No | Yes | No |
| Rosengren 1996[[32](#_ENREF_32)] | NFL | Yes | No |  | Yes | No | No | Alzheimer’s disease |
| Steinacker 2011[[33](#_ENREF_33)] | NFH | Yes | No |  | No | Tension headache | No | Parkinson disease |
| Steinacker 2015[[34](#_ENREF_34)] | NFH and NFL | Yes | No |  | No | Polyneuropathies  Facial Palsy | Yes | Alzheimer’s disease, Parkinson disease |
| Tortelli 2012[[35](#_ENREF_35)] | NFL | Yes | No |  | No | No | No | Chronic inflammatory demyelinating neuropathy, Alzheimer’s disease, mild cognitive impairment, spino-cerebellar ataxia, cortico basal degeneration, multi system atrophy |
| Tortelli 2014[[36](#_ENREF_36)] | NFL | Yes | No | longitudinal study) | N/A | N/A | N/A | N/A |
| Weydt 2016[[37](#_ENREF_37)] | NFH and NFL | Yes | Yes |  | Yes | No | No | No |
| Zetterberg 2007[[38](#_ENREF_38)] | NFL | Yes | No |  | Yes | No | No | headache, normal  pressure hydrocephalus, multifocal motor neuropathy,  spinobulbar muscular atrophy, Parkinson disease,  non-ALS dementias, multiple sclerosis and cerebrovascular  disease l |
