# Supplementary material for: Neurofilaments as Biomarkers for Amyotrophic Lateral Sclerosis: A Systematic Review and Meta-Analysis
Source: PLoS One. 2016 Oct 12;11(10):e0164625. doi: 10.1371/journal.pone.0164625 (PMC5061412; doi:10.1371/journal.pone.0164625)
Supplement: S3 Table — (DOCX) [file pone.0164625.s008.docx]

|  |  | CSF | Blood |
| --- | --- | --- | --- |
| NFH |  | Brettschneider et al. 2005  Reijn et al. 2008  Kuhle et al. 2010  Steinacker et al. 2011  Mendonca et al. 2011  Ganesalingam et al. 2011  Ganesalingam et al. 2013  Boylan et al. 2013 [[28](#_ENREF_28)]  Goncalves et al. 2014  Weydt et al. 2015  Lehnert et al. 2016  Steinacker et al. 2016 | Boylan et al.2009  Ganesalingam et al. 2011  Boylan et al. 2013  McCombe et al. 2015 |
| NFL |  | Rosengren et al. 1996  Zetterberg et al. 2007  Reijn et al. 2008  Tortelli et al. 2012  Gaiottino et al. 2013  Tortelli et al. 2014  Lu et al. 2015  Weydt et al. 2015  Steinacker et al. 2016 | Gaiottino et al. 2013  Lu et al. 2015  Weydt et al. 2015 |

Summarized result of the papers according to the biofluid type
