# Supplementary material for: Neurofilaments as Biomarkers for Amyotrophic Lateral Sclerosis: A Systematic Review and Meta-Analysis
Source: PLoS One. 2016 Oct 12;11(10):e0164625. doi: 10.1371/journal.pone.0164625 (PMC5061412; doi:10.1371/journal.pone.0164625)
Supplement: S4 Table — (DOCX) [file pone.0164625.s009.docx]

**Studies of correlation with duration**

|  | **The result of correlation** | **The method** | **P value** | **Number of patients** | **R value ( if use pearson or spearman correlation method)** |
| --- | --- | --- | --- | --- | --- |
| **Studies of NF Heavy chain** | | | | | |
| **CSF** | | | | | |
| Ganesalingam 2013 | Inverse correlation between CSF NFH and disease duration | Pearson’s correlation | P<0.0001 | 150 | -0.35 |
| Steinacker 2015 | Inverse correlation between CSF NFH and disease duration | Spearman’s correlation | P<0.0001 | 455 | -0.43 |
|  | No significant difference between the first test and the second follow up test in CSF NFH | Kruskal-Wallis test | P=0.520 | 11 | N/A |
| Weydt 2016 | Disease duration was significantly correlated with CSF NFH | Spearman’s correlation | P<0.0001 | 61 | -0.565 |
| **Blood** |  |  |  |  |  |
| Boylan 2009 | No correlation between baseline pNF-H and disease duration | Kendall’s tau correlation analysis | P=0.20 | 19 | N/A |
| **Studies of NF light chain** | | | | | |
| **CSF** | | | | | |
| Tortelli 2012 | There was negative correlation between CSF NFL and diagnostic delay ( time from onset to diagnosis) | Spearman’s correlation | P<0.0001 | 37 | -0.55 |
| Zetterberg 2007 | Inverse correlation between CSF NFL and disease duration | Spearman’s correlation | P=0.001 | 79 | -0.518 |
| Steinacker 2015 | Inverse correlation between CSF NFL and disease duration | Spearman’s correlation | P<0.0001 | 455 | -0.376 |
|  | There was significant difference between first test and the second follow up test in CSF NFL | Kruskal-Wallis test | P=0.007 | 11 | N/A |
| Wedyt 2016 | There was significant correlation between CSF NFL and disease duration | Spearman’s correlation | P<0.0001 | 51 | -0.606 |
| **Blood** | | | | | |
| Lu 2015 ( London cohort) | Negative correlation between blood NFL and disease duration | Correlation analysis | P=0.0002 | 108 | -0.36 |
| Lu 2015 ( Oxford cohort) | Negative correlation between blood NFL and disease duration | Correlation analysis | P<0.0001 | 64 | -0.5 |
| Wedyt 2016 | There was significant correlation between blood NFL and disease duration | Spearman’s correlation | P=0.012 | 51 | -0.401 |

**Studies of correlation with disease progression**

|  | **The result of correlation** | **The method** | **P value** | **Number of patients** | **R value ( if use pearson or spearman correlation method)** |
| --- | --- | --- | --- | --- | --- |
| **Studies of NF heavy chain** | | | | | |
| **CSF** | | | | | |
| Boylan 2012 ( Mayo 12 month Cohort) | There was negative correlation between CSF pNFH and ALSFRS-R change per month in the first 4 months follow up | Spearman’s correlation | P=0.006 | 20 | -0.59 |
|  | There was negative correlation between CSF pNFH and ALSFRS-R change per month in the first 12 months follow up | Spearman’s correlation | P=0.015 | 20 | -0.54 |
| Brettschneider 2006 | There was significant difference in the CSF pNFH between fast progression patients and slow progression patients. | Wilcoxon two samples test | P<0.001 | 49 | N/A |
| Steinacker 2011 | There was significant difference in the CSF pNFH between fast progression group and slow progression group | Mann-Whitney Rank sum test | P<0.001 | 68 | N/A |
| Steinacker 2015 | There was a weak tendency between CSF pNFH and ALSFRS-R | Spearman’s correlation | P=0.016 | 455 | -0.163 |
|  | There is significant difference between fast progression group and slow progression group | Kruskal-Wallis test | P=0.026 | 455 | N/A |
| Weydt 2016 | Disease progression was not correlated with CSF NFH | Spearman’s correlation | P=0.27 | 61 | -0.285 |
| **Blood** |  |  |  |  |  |
| McCombe 2015 | There was no relationship between the serum pNFH and ALSFRS-R | Linear regression analysis | P>0.05 | 98 | N/A |
|  | There was an inverse relationship between the serum pNFH and ALSFRS/days×10^3^ | Linear regression analysis | P=0.005 | 98 | N/A |
|  | There was an inverse relationship between the rate of rise in serum pNFH and ALSFRS/days×10^3^ | Linear regression analysis | P=0.0635 | 98 | N/A |
| Boylan 2009 | There was no correlation between baseline plasma pNFH and ALSFRS-R | Kendall’s tau correlation analysis | P=0.44 | 19 | N/A |
|  | There was weak correlation between baseline plasma pNFH and ALSFRS-R decline over 4 months | Kendall’s tau correlation analysis | P=0.087 | 19 | N/A |
| Boylan 2012 ( Mayo 12 month cohort) | There was negative correlation between serum pNFH and ALSFRS-R change per month In the first 4 months follow up | Spearman’s correlation | P=0.042 | 20 | -0.46 |
|  | There was no significant negative correlation between serum pNFH and ALSFRS-R change per month in the first 12 months follow up | Spearman’s correlation | P=0.19 | 20 | -0.31 |
|  | There was significant negative correlation between plasma pNFH and ALSFRS-R change per month In the first 4 months follow up | Spearman’s correlation | P=0.03 | 38 | -0.35 |
|  | There was no negative correlation between plasma pNFH and ALSFRS-R change per month In the first 12 months follow up | Spearman’s correlation | P=0.29 | 20 | -0.25 |
| **Studies of light chain** | | | | | |
| **CSF** | | | | | |
| Tortelli 2012 | There was negative correlation between CSF NFL and ALSFRS-R | Spearman’s correlation | P=0.014 | 37 | -0.41 |
|  | There was positive correlation between CSF NFL and progression rate | Spearman’s correlation | P=0.65 | 37 | 0.65 |
| Steinacker 2015 | There was a weak tendency between CSF NFL and ALSFRS-R | Spearman’s correlation | P=0.013 | 455 | -0.016 |
|  | There was significant difference in the CSF NFL between fast progression group and slow progression group | Kruskal-Wallis test | P=0.47 | 455 | N/A |
| Lu 2015 ( oxford cohort) | The fast progression patients’ CSF NFL concentration was significantly higher than slow progression patients | Non parametric analysis | P=0.005 | 64 | N/A |
| Wedyt 2016 | There was no significant correlation between CSF NFL and ALSFRS-R | Spearman’s correlation | P=0.46 | 51 | -0.199 |
| **Blood** |  |  |  |  |  |
| Lu 2015 ( London cohort) | The fast progression patients’ blood NFL concentration was significantly higher than slow progression patients | Non parametric analysis | P=0.0002 | 103 | N/A |
| Lu 2015 ( Oxford cohort) | The fast progression patients’ blood NFL concentration was significantly higher than slow progression patients | Non parametric analysis | P=0.0007 | 64 | N/A |
| Wedyt 20116 | There was no significant correlation between blood NFL and ALSFRS-R | Spearman’s correlation | P=0.14 | 51 | -0.359 |

**Studies of correlation with survival time**

|  | **The result of correlation** | **The method** | **P value** | **Number of patients** | **R value ( if use pearson or spearman correlation method)** |
| --- | --- | --- | --- | --- | --- |
| **Studies of NF heavy chain** | | | | | |
| **CSF** | | | | | |
| Boylan 2012 ( Mayo 12 month cohort) | Doubling in CSF pNFH caused 1.80 fold increase of hazard ratio of death ( time from sample) | Cox proportional hazards regression model | P=0.12 | 20 | N/A |
|  | Doubling in CSF pNFH caused 1.80 fold increase of hazard ratio of death ( time from onset) | Cox proportional hazards regression model | P=0.14 | 20 | N/A |
| Steinacker 2015 | There is significant difference in the survival time among the patients with low, intermediate and high concentration CSF NFH | Kaplan meier survival curve | P=0.0024 | 45 | N/A |
| **Blood** |  |  |  |  |  |
| McCombe 2015 | There was an inverse correlation between pNFH level and survival time | Linear regression analysis | P=0.0481 | 98 | N/A |
|  | There was an inverse correlation between the rate of rise of pNFH and survival time | Linear regression analysis | P=0.0247 | 98 | N/A |
| Boylan 2009 | There was no association between cumulative mortality and base line pNFH | Kaplan meier survival curve | P=0.21 | 19 | N/A |
| Boylan 2012 ( Mayo 12 month cohort plus Mayo 4 month cohort plus Emory cohort) | Doubling in plasma pNFH caused 1.32 fold increase of hazard ratio of death ( time from sample) | Cox proportional hazards regression model | P=0.012 | 62 | N/A |
|  | Doubling in plasma NFH caused 1.18 fold increase of hazard ratio of death ( time from onset) | Cox proportional hazards regression model | P=0.11 | 62 | N/A |
| **Studies of Light chain** | | | | | |
| **CSF** | | | | | |
|  |  |  |  |  |  |
| Tortelli 2013 | The ALS patients’ survival time with high CSF NFL was significant reduced | Kaplan meier survival curve | P<0.0001 | 37 | N/A |
| Zetterberg 2007 | CSF NFL was inversely correlated with survival time | Spearman’s correlation | P=0.001 | 79 | -0.518 |
|  | The ALS patients with high CSF NFL had short survival time compared with the ALS patients with low CSF NFL | Kaplan meier survival curve | P=0.002 | 79 | N/A |
| Steinacker 2015 | There is significant difference in the survival time among the patients with low, intermediate and high concentration CSF NFL | Kaplan meier survival curve | P=0.0261 | 45 | N/A |
| Lu 2015 ( London cohort plus Oxford cohort) | NFL in CSF was associated poor survival | Cox proportional hazards regression model | P=0.002 | 12 | N/A |
| **Blood** | | | | | |
| Lu 2015 ( London cohort plus Oxford cohort) | NFL in blood was associated poor survival | Cox proportional hazards regression model | P<0.001 | 33 | N/A |
