# Supplementary material for: Neurofilaments as Biomarkers for Amyotrophic Lateral Sclerosis: A Systematic Review and Meta-Analysis
Source: PLoS One. 2016 Oct 12;11(10):e0164625. doi: 10.1371/journal.pone.0164625 (PMC5061412; doi:10.1371/journal.pone.0164625)
Supplement: S5 Table — (DOCX) [file pone.0164625.s010.docx]

ALS versus healthy controls/without parenchymal CNS involvement in CSF NFH

| Study name | Cut off value  (ng/ml) | Sensitivity | Specificity | Area under curve ( AUC) |
| --- | --- | --- | --- | --- |
| Brettschneider 2006 | 0.95 | 0.71 | 0.88 | 0.87 |
| Weydt 2016 | N/A | N/A | N/A | N/A |
| Steinacker 2011 | N/A | N/A | N/A | N/A |
| Kuhle 2010 | N/A | N/A | N/A | N/A |
| Steinacker 2016 | 0.56 | 0.83 | 0.80 | 0.871 |

ALS versus ALS mimics disease in CSF NFH

| Study name | Cut off value  (ng/ml) | Sensitivity | Specificity | Area under curve (AUC) |
| --- | --- | --- | --- | --- |
| Steinacker 2016 | 0.56 | 0.83 | 0.80 | 0.841 |
| Reijn 2009 | 0.502 | 0.72 | 0.80 | 0.82 |

ALS versus other neurological diseases with CNS involvement in CSF NFH

| Study name | Cut off value  (ng/ml) | Sensitivity | Specificity | Area under curve ( AUC) |
| --- | --- | --- | --- | --- |
| Brettschneider 2006 | 0.41 | 0.89 | 0.92 | 0.94 |
| Lehnert 2014 | N/A | N/A | N/A | N/A |
| Steinacker 2011 | N/A | N/A | N/A | N/A |
| Kuhle 2010 | N/A | N/A | N/A | N/A |
| Steinacker 2016 | 0.56 | 0.83 | 0.80 | 0.871 |
| Goncalves 2015 | 0.385 | 0.828 | 0.647 | 0.8590 |

ALS versus healthy controls/without parenchymal CNS involvement in blood NFH

| Study name | Cut off value  (ng/ml) | Sensitivity | Specificity | Area under curve ( AUC) |
| --- | --- | --- | --- | --- |
| McCombe 2015 | N/A | N/A | N/A | N/A |
| Boylan 2009 | N/A | N/A | N/A | N/A |

ALS versus healthy controls/without parenchymal CNS involvement in CSF NFL

| Study name | Cut off value  (ng/ml) | Sensitivity | Specificity | Area under curve ( AUC) |
| --- | --- | --- | --- | --- |
| Gaiottino 2013 | N/A | N/A | N/A | N/A |
| Rosengren 1996 | N/A | N/A | N/A | N/A |
| Steinacker 2016 | 2.2 | N/A | N/A | 0.871 |
| Lu 2015 | 1.781 | 0.97 | 0.95 | 0.9987 |
| Weydt 2016 | N/A | N/A | N/A | N/A |
| Zetterberg 2007 | N/A | N/A | N/A | N/A |

ALS versus ALS mimics disease in CSF NFL

| Study name | Cut off value  (ng/ml) | Sensitivity | Specificity | Area under curve ( AUC) |
| --- | --- | --- | --- | --- |
| Reijn 2009 | 22.6 | 0.75 | 0.79 | 0.77 |
| Steinacker 2016 | 2.2 | 0.77 | 0.88 | 0.841 |

ALS versus other neurological diseases with CNS involvement in CSF NFL

| Study name | Cut off value  (ng/ml) | Sensitivity | Specificity | Area under curve ( AUC) |
| --- | --- | --- | --- | --- |
| Gaiottino 2013 | N/A | N/A | N/A | N/A |
| Rosengren 1996 | N/A | N/A | N/A | N/A |
| Steinacker 2011 | N/A | N/A | N/A | N/A |
| Tortelli 2012 | 1.981 | 0.784 | 0.725 | 0.79 |
| Zetteberg 2007 | N/A | N/A | N/A | N/A |

ALS versus healthy controls/without parenchymal CNS involvement in blood NFL

| Study name | Cut off value  (ng/ml) | Sensitivity | Specificity | Area under curve ( AUC) |
| --- | --- | --- | --- | --- |
| Gaiottino 2013 | 0.026 | 0.913 | 0.91 | N/A |
| Lu 2015( oxford cohort) | 0.036 | 0.89 | 0.75 | 0.8626 |
| Lu 2015 (londone cohort) | 0.0362 | 0.90 | 0.71 | 0.8687 |
| Weydt 2016 | N/A | N/A | N/A | N/A |
